# SIRT7 deficiency suppresses inflammation, induces EndoMT, and increases vascular permeability in primary pulmonary endothelial cells

**DOI:** 10.1038/s41598-020-69236-z

**Published:** 2020-07-27

**Authors:** Anne E. Wyman, Trang T. T. Nguyen, Pratap Karki, Mohan E. Tulapurkar, Chen-Ou Zhang, Junghyun Kim, Theresa G. Feng, Abdoulaye J. Dabo, Nevins W. Todd, Irina G. Luzina, Patrick Geraghty, Robert F. Foronjy, Jeffrey D. Hasday, Anna A. Birukova, Sergei P. Atamas, Konstantin G. Birukov

**Affiliations:** 1 Geriatric Research Education and Clinical Center (GRECC), VA Maryland Health Care System, Baltimore VA Medical Center, Baltimore, MD USA; 2 Department of Medicine, University of Maryland School of Medicine, Baltimore, MD USA; 3 Research Service, Baltimore VA Medical Center, Baltimore, MD USA; 4 Departments of Medicine and Cell Biology, SUNY Downstate Health Sciences University, Brooklyn, NY USA; 5 Department of Anesthesiology, SUNY Downstate Health Sciences University, Brooklyn, NY USA

**Keywords:** RHO signalling, Mechanisms of disease

## Abstract

Acute lung injury (ALI), a common condition in critically ill patients, has limited treatments and high mortality. Aging is a risk factor for ALI. Sirtuins (SIRTs), central regulators of the aging process, decrease during normal aging and in aging-related diseases. We recently showed decreased SIRT7 expression in lung tissues and fibroblasts from patients with pulmonary fibrosis compared to controls. To gain insight into aging-related mechanisms in ALI, we investigated the effects of SIRT7 depletion on lipopolysaccharide (LPS)-induced inflammatory responses and endothelial barrier permeability in human primary pulmonary endothelial cells. Silencing SIRT7 in pulmonary artery or microvascular endothelial cells attenuated LPS-induced increases in ICAM1, VCAM1, IL8, and IL6 and induced endomesenchymal transition (EndoMT) with decreases in VE-Cadherin and PECAM1 and increases in collagen, alpha-smooth muscle actin, TGFβ receptor 1, and the transcription factor Snail. Loss of endothelial adhesion molecules was accompanied by increased F-actin stress fibers and increased endothelial barrier permeability. Together, these results show that an aging phenotype induced by SIRT7 deficiency promotes EndoMT with impaired inflammatory responses and dysfunction of the lung vascular barrier.

## Introduction

Acute lung injury (ALI) and its more severe form, acute respiratory distress syndrome (ARDS), occur commonly in critically ill patients and result in high mortality. ARDS, acute onset respiratory failure with bilateral lung infiltrates and severe hypoxemia, can result from direct lung insults such as gastric aspiration or pneumonia, or indirect injury from sepsis or trauma^1^. Pneumonia and sepsis are the most common causes of ALI and ARDS^2,3^. Disruption and increased permeability of alveolar and endothelial barriers in ARDS result in transendothelial migration of fluid, proteins, and inflammatory cells into the alveolar space^2,4^. Despite progress in understanding mechanisms for the development and resolution of lung injury^2^, mortality remains high at 30–50%^3,5,6^. Pharmacologic treatments are ineffective, and supportive management with lung protective ventilation remains the mainstay of therapy^7^.

Susceptibility to lung disease increases with age^8–10^. Aging is a risk factor for ALI and its leading cause, sepsis, in multiple population-based studies^6,11–16^. Aging-related processes such as dysregulated inflammatory responses^17,18^, altered metabolic pathways^19^, propensity for thrombosis^20^, increased oxidative stress^21,22^, proteostasis imbalance^23^, and stem cell dysfunction^24^ have recently been implicated in murine and human cell culture models of sepsis and ALI. However, the precise molecular mechanisms for the increased risk and worse outcomes of ALI in elderly patients remain poorly understood.

Sirtuins (SIRTs), a family of histone deacetylases that require nicotinamide adenine dinucleotide (NAD^+^) for their catalytic activity, are among central regulators of the aging process^25^. SIRTs protect against aging through diverse mechanisms including regulation of metabolism, mitochondrial maintenance, DNA repair, telomere stability, and autophagy^26,27^. SIRTs decrease during normal aging, and their loss is associated with numerous aging-related diseases such as neurodegenerative, cardiovascular, and metabolic disorders^28–34^. More recently, studies have emerged on the role of SIRTs in fibrotic skin and lung diseases, particularly systemic sclerosis (SSc) and idiopathic pulmonary fibrosis (IPF) (see^35^ for a review). We investigated the expression of all seven mammalian SIRTs in lung tissues and fibroblasts from patients with SSc and IPF and found a tendency for SIRTs to be decreased in patients with fibrosis compared to controls, with a particularly notable decline in SIRT7^36^. Similar to other studies on SIRT1 and SIRT3^37–41^, SIRT7 overexpression in human lung fibroblasts had an antifibrotic effect, suppressing basal and TGFβ-induced increases in collagen and alpha smooth muscle actin (αSMA)^36^.

Activation of SIRT1 and SIRT3 have been shown to protect against endotoxin-induced vascular dysfunction and acute lung injury^42–47^. However, the role of SIRT7 in ALI has not been explored. Furthermore, how SIRTs regulate endothelial cell barrier function, at baseline and in response to barrier disruptive agonists, remains unclear. To gain insight into aging-related mechanisms of ALI, we investigated the relationship between SIRT7 suppression, acute inflammatory and fibrotic responses, and endothelial barrier function in LPS-induced models of ALI.

## Results

### SIRT7 loss occurs during aging and LPS exposure and is associated with inflammation and fibrosis in murine lung tissues in vivo

**Figure 1:**
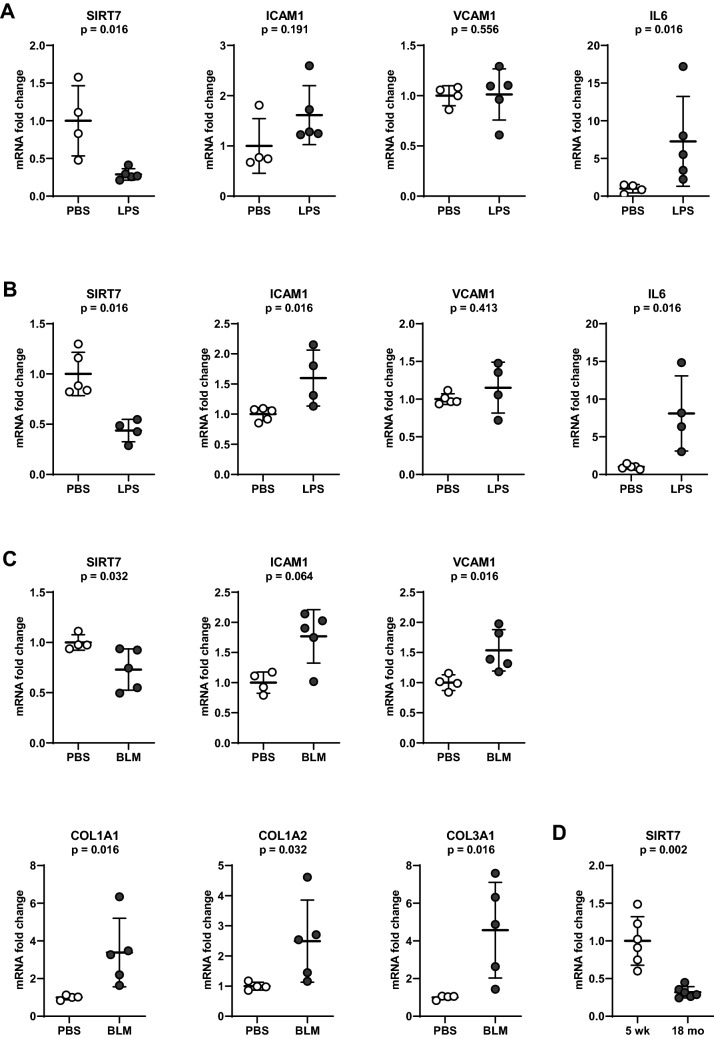
Effects of age, LPS, or bleomycin on mRNA levels of SIRT7 and pro-inflammatory and pro-fibrotic markers measured by RT-qPCR in mouse lung tissues. ICAM1, VCAM1, IL6, and SIRT7 mRNA levels normalized to respective β-actin mRNA levels in lung tissue samples of C57BL/6 (**A**) or A/J mice (**B**) 24 h after intranasal treatment with LPS or PBS. (**C**) ICAM1, VCAM1, SIRT7, COL1A1, COL1A2, and COL3A1 mRNA levels normalized to respective 18S rRNA levels in lung tissue samples of C57BL/6 mice 14 days after intra-tracheal instillation of bleomycin or PBS. (**D**) SIRT7 mRNA levels relative to GAPDH in 5 week- and 18 month-old C57BL/6 mice. Data are expressed as fold changes relative to average mRNA levels in control (PBS-treated) mice in (**A**–**C**) and fold changes relative to average mRNA value in 5 week-old mice in (**D**). Each circle represents a separate tested animal. ○ Control (PBS-treated mice), ● LPS- or bleomycin-challenged mice. Means and standard deviations are shown. *P* values were calculated using the Mann–Whitney *U-*test.

To evaluate SIRT7 expression and its association with pro-inflammatory responses typical of acute lung injury in vivo, mRNA levels of SIRT7, ICAM1, VCAM1, and IL6 were measured in lung tissues from C57BL/6 (Fig. 1A) and A/J (Fig. 1B) mice administered LPS or saline for 24 h. LPS induced a significant decrease in SIRT7 mRNA levels in both groups of mice and a pro-inflammatory response with increases in ICAM1 and IL6 in C57BL/6 mice and increases in ICAM1, VCAM1, and IL6 in A/J mice. To model the fibrotic stage of ALI, C57/BL/6 mice were administered intratracheal bleomycin or PBS for 14 days. Bleomycin induced a significant decrease in SIRT7 mRNA levels in murine lung tissues, as shown previously^36^, with corresponding increases in ICAM1, VCAM1, and collagen chains COL1A1, COL1A2, and COL3A1 (Fig. 1C). To evaluate the effect of aging on SIRT7 levels, SIRT7 mRNA levels were measured in lung tissues from six 5 week-old and six 18 month-old C57BL/6 mice. SIRT7 mRNA levels were significantly lower in lung tissues from aged compared to young mice with a threefold decrease at age 18 months compared to 5 weeks (Fig. 1D).

### SIRT7 silencing suppresses LPS-induced pro-inflammatory responses in primary pulmonary endothelial cells

**Figure 2:**
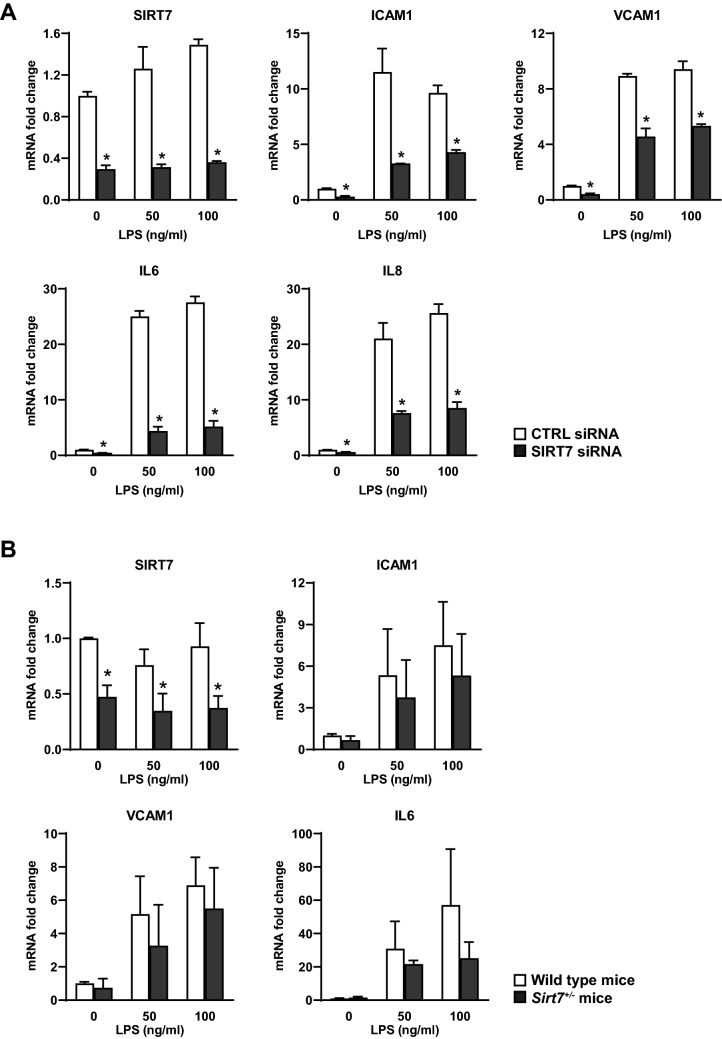
Effects of SIRT7 silencing on mRNA levels of inflammatory mediators in human and murine primary pulmonary endothelial cells. SIRT7, ICAM1, VCAM1, IL6, or IL8 mRNA levels were measured by RT-qPCR and normalized to GAPDH mRNA levels in HPAEC cultures (**A**) or to β-actin mRNA levels in endothelial cell cultures from wild type or *Sirt7*^+*/−*^ mice (**B**). Data are expressed as fold changes relative to average mRNA levels in unstimulated (LPS 0) cell cultures transfected with CTRL siRNA (**A**) or unstimulated cell cultures from wild type mice (**B**). In (**A**), bars represent averages and brackets standard deviations of measurements for two samples per condition for CTRL-silenced (□) and SIRT7-silenced (■) cell cultures. Experiments were performed on at least three separate occasions with similar results. In (**B**), bars represent averages and brackets standard deviations of cell cultures from three wild type (□) and three *Sirt7*^+*/−*^ (■) mice. Stars denote significant differences (*P* < 0.05) between CTRL- and SIRT7-silenced or wild type and *Sirt7*^+*/−*^ endothelial cell cultures at each LPS concentration. *P* values were calculated using the Student’s *t* test.

Given the heterogeneity of lung tissue, which contains diverse cell types, and considering the central role of the pulmonary vasculature in ALI, our next experiments focused on the effects of SIRT7 loss on inflammatory responses in cultured primary pulmonary endothelial cells. Transfection of human pulmonary artery endothelial cell (HPAEC) cultures with SIRT7 siRNA, compared to CTRL siRNA, resulted in three to four-fold decreases in SIRT7 mRNA expression. Stimulation with LPS induced increases in ICAM1, VCAM1, IL6, and IL8 mRNA levels in both CTRL- and SIRT7-silenced HPAEC after 6 h. However, compared to CTRL-silenced cultures, SIRT7 silencing resulted in significant decreases in ICAM1, VCAM1, IL6, and IL8 mRNA levels both under basal, unstimulated conditions as well as in response to LPS (Fig. 2A).

**Figure 3:**
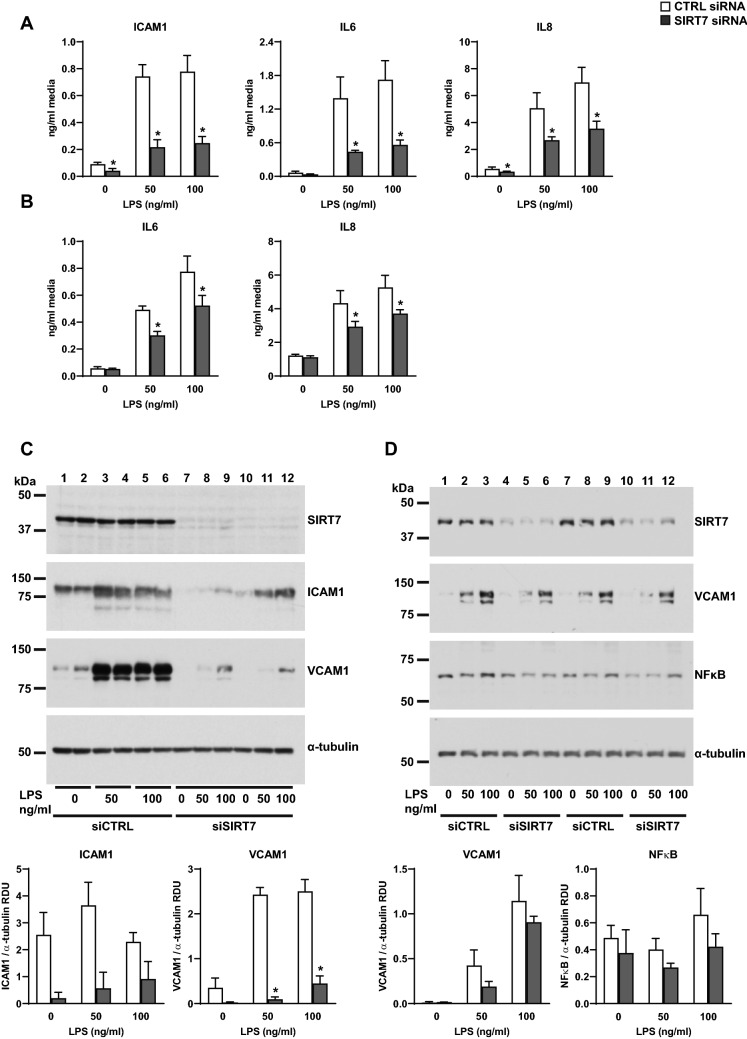
Effects of SIRT7 silencing on protein levels of inflammatory mediators in human primary pulmonary endothelial cells. ELISA measurements of soluble ICAM1, IL6, or IL8 protein levels in media from CTRL- or SIRT7 silenced HPAEC (**A**) and HPMVEC (**B**). WB measurements of SIRT7, ICAM1, VCAM1, or total NFκB protein levels in cell lysates from CTRL- or SIRT7-silenced HPAEC (**C**) and HPMVEC (**D**). Data shown are for two replicate samples per condition for CTRL siRNA and one sample per condition for two different strands of SIRT7 siRNA. Different proteins for the same group of samples, either from the same membrane or different gel and membrane, are demarcated by white spaces. Full-length blots are presented in Supplementary Figures 1 and 2. Densitometry values for ICAM1 and VCAM1 (**C**) and VCAM and NFκB (**D**) relative to α-tubulin are shown below the respective WB. In all panels, bars represent averages and brackets standard deviations of measurements for two samples per condition for CTRL-silenced (□) and SIRT7-silenced (■) cell cultures. Stars denote significant differences (*P* < 0.05) between CTRL- and SIRT7-silenced cell cultures at each LPS concentration. Experiments were performed on at least three separate occasions with similar results. *P* values were calculated using the Student’s *t* test.

Similar responses were found in endothelial cells isolated from mice heterozygous for *Sirt7 (Sirt7*^+*/−*^*)*. Compared to wild type mice, *Sirt7*^+*/−*^ mice expressed two to three-fold less SIRT7 mRNA and showed attenuated increases in ICAM1, VCAM1, and IL6 mRNA levels in response to LPS stimulation (Fig. 2B). Similarly, SIRT7 silencing in HPAEC resulted in significant decreases in soluble ICAM1, IL6, and IL8 protein levels in LPS-conditioned media (Fig. 3A) and attenuated LPS-induced increases in IL6 and IL8 in human pulmonary microvascular endothelial cells (HPMVEC) (Fig. 3B). SIRT7 silencing also decreased basal and LPS-induced increases in ICAM1 and VCAM1 protein levels in HPAEC whole cell lysates (Fig. 3C, Supplementary Figure 1) and LPS-induced increases in VCAM1 in HPMVEC (Fig. 3D, Supplementary Figure 2). We did not detect differences in IL6 or IL8 between CTRL- and SIRT7-silenced HPMVEC cultures under basal, unstimulated conditions (Fig. 3B) or differences in ICAM1 protein levels between CTRL- and SIRT7-silenced HPMVEC (data not shown). Although LPS stimulation suppressed SIRT7 mRNA levels in murine lung tissues in vivo (Fig. 1), we did not observe a consistent change in SIRT7 mRNA levels in response to LPS in human or murine endothelial monolayers (Fig. 2). To investigate the contribution of other pulmonary cell types to this discrepancy, primary human small airway epithelial cells (SAEC) were stimulated with LPS, which resulted in a significant, approximately twofold, decrease in SIRT7 mRNA levels after 24 h (Supplementary Figure 3A). In contrast to endothelial cell cultures, SIRT7 suppression with siRNA in SAEC cultures resulted in pro-inflammatory effects with significant increases in IL6 and IL8 mRNA levels (Supplementary Figure 3B). Taken together, these data suggest that LPS-induced pro-inflammatory effects in vivo are due, at least in part, to LPS-induced SIRT7 suppression in airway epithelium.

### SIRT7 silencing suppresses NFκB signaling in human primary pulmonary endothelial cells

**Figure 4:**
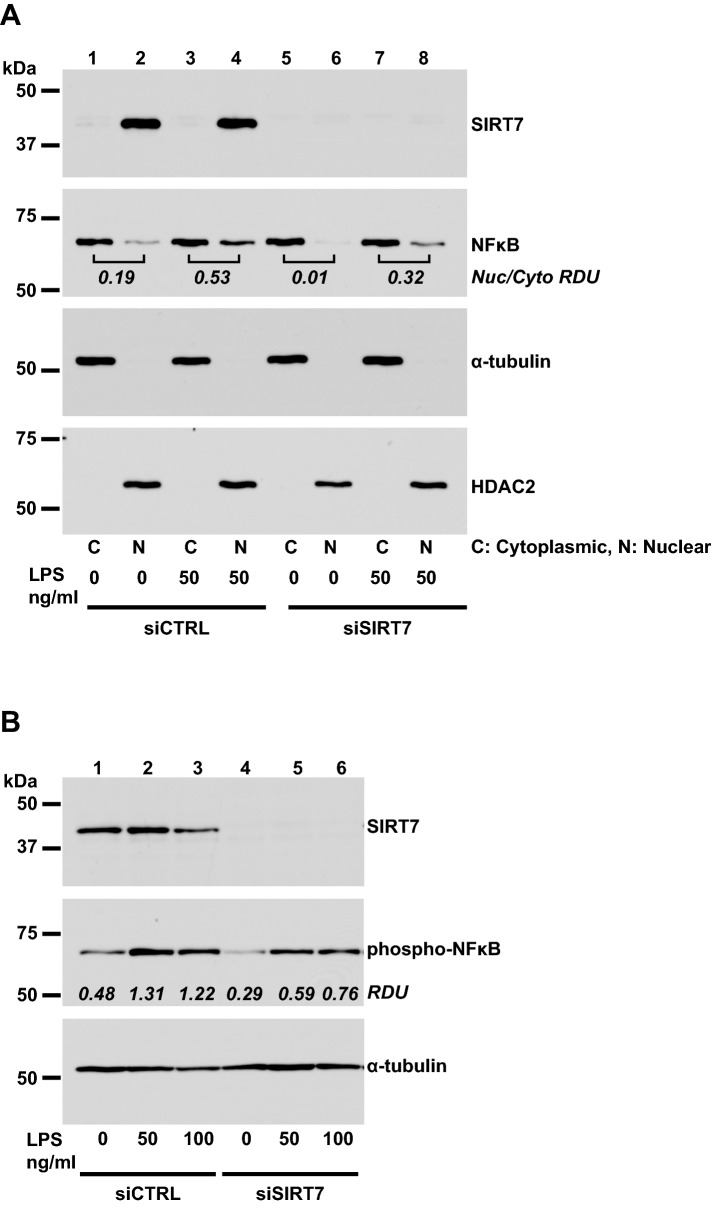
Effects of SIRT silencing on NFκB signaling. (**A**) Total NFκB protein in cytoplasmic (“C”) and nuclear (“N”) fractions of CTRL- or SIRT7-silenced HPAEC 6 h after LPS stimulation. α-tubulin and HDAC2 were used to control for sample loading of cytoplasmic and nuclear fractions, respectively. Ratios of nuclear to cytoplasmic NFκB for each condition are designated in the panel for NFκB. (**B**) Phosphorylated NFκB protein levels in CTRL- or SIRT7-silenced HPAEC 2 h after LPS stimulation. Densitometry values for phosphorylated NFκB relative to α-tubulin (RDU) are shown. Different proteins for the same group of samples are demarcated by white spaces.

To investigate possible mechanisms for the observed suppression of LPS-induced inflammatory responses in pulmonary endothelial cells, phosphorylated and total NFκB levels were measured in whole cell lysates and nuclear fractions from CTRL- or SIRT7-silenced endothelial cell cultures. Silencing SIRT7 in HPMVEC resulted in decreased total NFκB protein levels in whole cell lysates under basal, unstimulated conditions and 6 h after stimulation with LPS (Fig. 3D, Supplementary Figure 2). SIRT7 silencing in HPAEC decreased total NFκB levels in nuclear fractions of basal and LPS-stimulated cultures 6 h after LPS treatment (Fig. 4A, Supplementary Figure 4). SIRT7 silencing in HPAEC also attenuated basal and LPS-induced increases in phosphorylated NFκB 2 h after LPS stimulation (Fig. 4B, Supplementary Figure 5).

**Figure 5:**
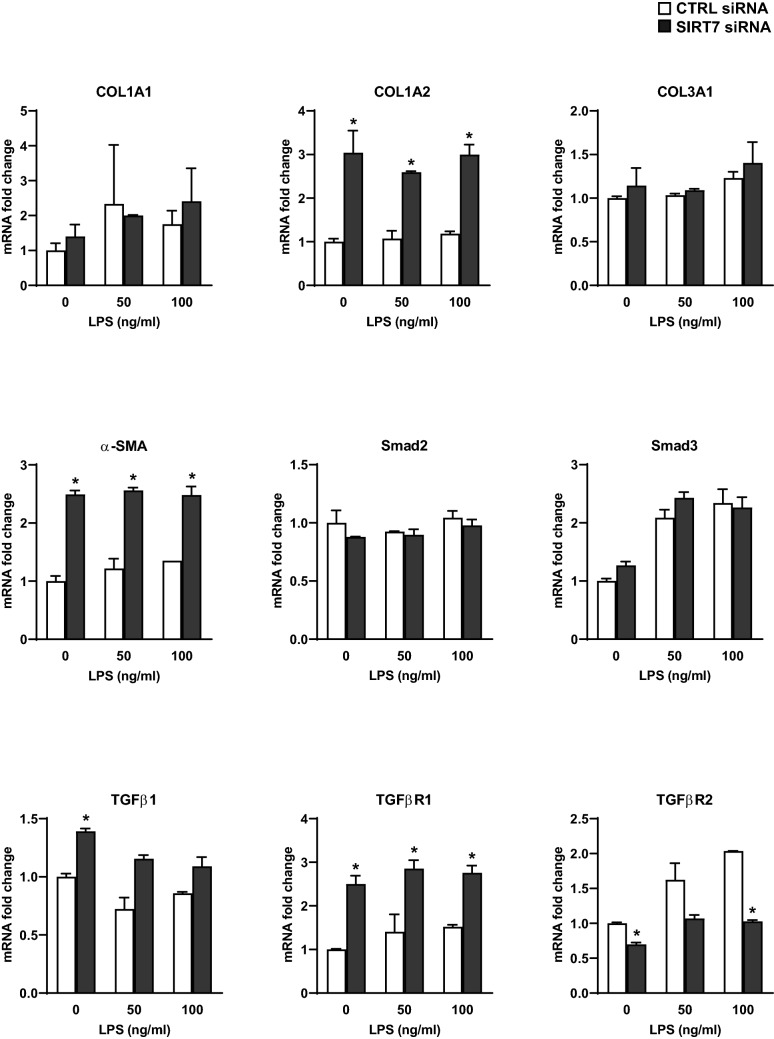
Effects of SIRT7 silencing on pro-fibrotic gene expression in primary pulmonary endothelial cells. mRNA levels of each indicated RT-qPCR target in CTRL-silenced (□) or SIRT7-silenced (■) HPAEC cultures were normalized to GAPDH and expressed as fold changes relative to average mRNA levels in unstimulated (LPS 0), CTRL-silenced cell cultures. Bars represent averages and brackets standard deviations of measurements for two samples per condition. *P* values were calculated using the Student’s *t* test. Significant differences (*P* < 0.05) between and CTRL- and SIRT7-silenced cultures for each LPS concentration are denoted by stars.

### SIRT7 silencing induces EndoMT in pulmonary endothelial cells through regulation of the TGFβ signaling pathway

We observed that SIRT7-silenced endothelial cell cultures appeared to have a more elongated, mesenchymal-appearing morphology when viewed under the light or fluorescent microscope (Supplementary Figure 6). This observation suggested that SIRT7 suppression may be inducing EndoMT in endothelial cells. To investigate this finding further, mRNA isolated from CTRL- or SIRT7-silenced HPAEC was tested for mesenchymal markers by RT-qPCR. Silencing SIRT7 in HPAEC resulted in significant increases in COL1A2 and α-SMA mRNA levels under basal conditions and after stimulation with LPS for 6 h (Fig. 5). COL1A2 was the most highly expressed of the three collagen chains tested as well as the one most affected by SIRT7 silencing and remained significantly elevated in SIRT7-silenced cultures up to 48 h after stimulation with LPS (Supplementary Figure 7). Silencing SIRT7 also resulted in significant increases in TGFβ receptor 1 (TGFβR1) with smaller increases in TGFβ1 and decreases in TGFβ receptor 2 (TGFβR2) mRNA levels under basal and LPS-stimulated conditions (Fig. 5). Decreased TGFβR2 levels in SIRT7-silenced cultures may be mediated by negative feedback from increased TGFβ1 levels, as previously observed^36^.

**Figure 6:**
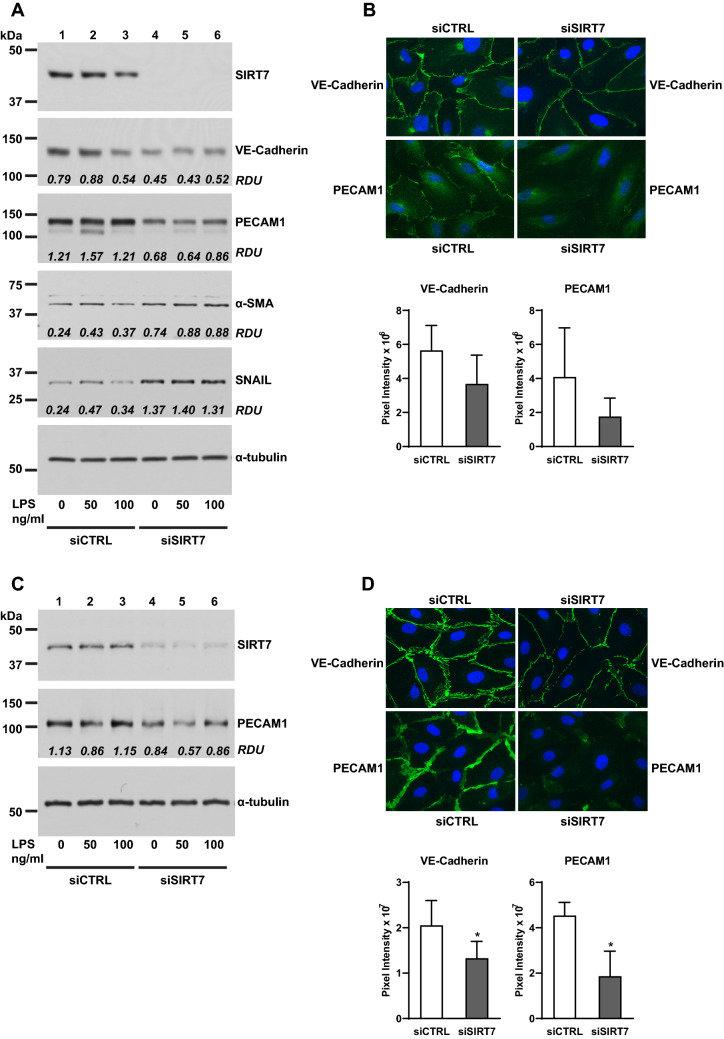
Effects of SIRT7 silencing on EndoMT in primary pulmonary endothelial cells. (**A**) WB for SIRT7, VE-Cadherin, PECAM1, α-SMA, and Snail in CTRL- or SIRT7-silenced HPAEC. Densitometry values (RDU) for VE-Cadherin relative to GAPDH (shown in Supplementary Figure 8C), PECAM1 relative to relative to α-tubulin, and α-SMA and Snail relative to GAPDH (Supplementary Figure 8H) are shown in each panel. (**B**) Representative IF images for VE-Cadherin and PECAM1 in CTRL- or SIRT7-silenced HPAEC. Bars represent average pixel intensities and brackets standard deviations of 6 images per condition. (**C**) WB for SIRT7 and PECAM1 in CTRL- or SIRT7-silenced HPMVEC. Densitometry values for PECAM1 relative to α-tubulin (RDU) are shown. (**D**) IF for VE-Cadherin and PECAM1 in CTRL- or SIRT7-silenced HPMVEC. Bars represent average pixel intensities and brackets standard deviations of 6 images per condition. Significant differences (*P* < 0.05) are denoted by stars. *P* values were calculated using the Student’s *t*-test. In (**A**,**C**), different proteins for the same group of samples are demarcated by white spaces.

**Figure 7:**
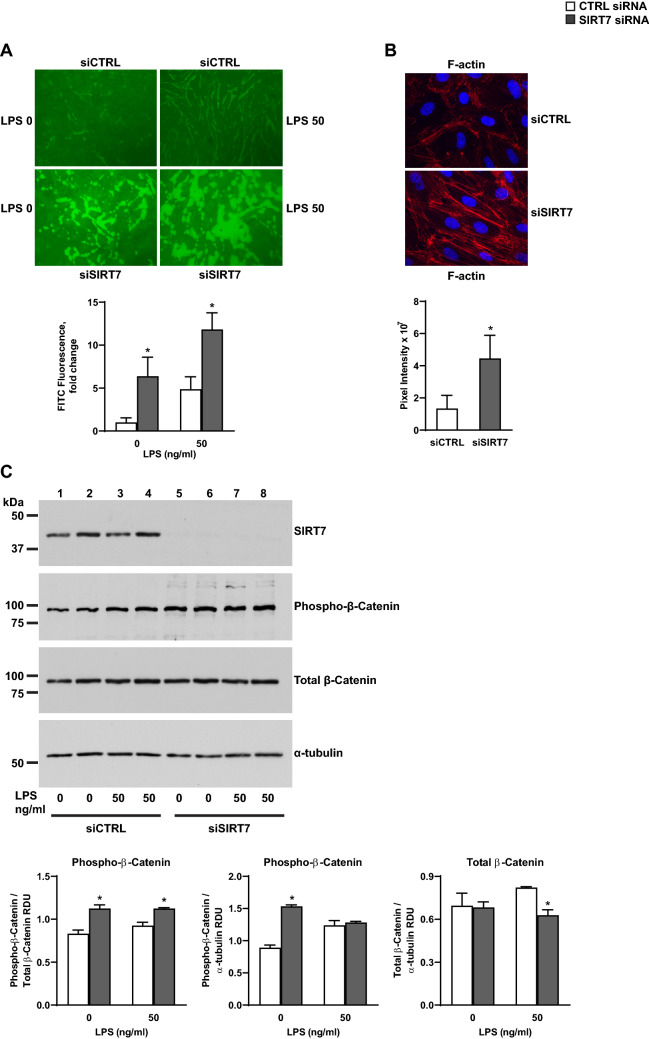
Effects of SIRT7 silencing on barrier permeability in endothelial cell cultures. (**A**) Effect of SIRT7 silencing in HPAEC on endothelial barrier permeability measured by XPerT assays 6 h after stimulation with LPS 50 µg/ml or no stimulus. Average fluorescence values and standard deviations of 6 images expressed as fold changes relative to the average value of unstimulated, CTRL-silenced cultures are shown below the image. (**B**) Representative IF images for F-actin stress fibers in CTRL- or SIRT7-silenced HPAEC 48 h after transfection. Average pixel intensities and standard deviations of 6 images per condition are shown. (**C**) Phosphorylated and total β-Catenin protein levels 6 h after LPS stimulation or no treatment in CTRL-silenced (□) or SIRT7-silenced (■) HPAEC cultures. Densitometry measurements of phosphorylated β-Catenin relative to total β-Catenin or α-tubulin (also shown in Supplementary Figure 11E) and total β-Catenin relative to α-tubulin (shown in Supplementary Figure 11C) are shown. Each bar represents the average of two separate samples per condition. Significant differences (*P* < 0.05) are denoted by stars. *P* values were calculated using the Student’s *t* test. For (**C**), different proteins for the same group of samples are demarcated by white spaces.

Silencing SIRT7 in HPAEC induced EndoMT as demonstrated by lower levels of the endothelial cell junction proteins VE-Cadherin and PECAM1 by WB (Fig. 6A, Supplementary Figure 8) and IF (Fig. 6B) and higher protein levels of α-SMA and the transcription factor Snail (Fig. 6A, Supplementary Figure 8). Similar to findings in HPAEC, silencing SIRT7 in HPMVEC resulted in lower VE-Cadherin and PECAM1 protein levels as assessed by WB (Fig. 6C, Supplementary Figure 9) and IF (Fig. 6D). We did not detect any collagen protein by WB or α-SMA protein by IF in endothelial cell cultures (data not shown).

### SIRT7 silencing increases endothelial barrier permeability in primary pulmonary endothelial cells

To investigate the effects of SIRT7 silencing on endothelial barrier permeability, Electric cell-substrate impendence sensing (ECIS) and Express Micromolecule Permeability Testing Assays (XPerT) were performed in CTRL- and SIRT7-silenced endothelial cell cultures. We did not detect any differences in basal endothelial permeability by ECIS between CTRL- and SIRT7-silenced HPAEC cultures or after cell stimulation with LPS or thrombin. Both experimental groups responded by increased permeability to LPS and thrombin stimulation (Supplementary Figure 10). However, SIRT7- compared to CTRL-silenced HPAEC cultures exhibited a significant increase in endothelial barrier permeability by XPerT assays, an effect exacerbated by LPS (Fig. 7A). Silencing SIRT7 in HPAEC increased F-actin stress fibers (Fig. 7B) and phosphorylation of the cellular adhesion protein β-Catenin at a residue which targets β-Catenin for destruction in the cytoplasm (Fig. 7C, Supplementary Figure 11). Silencing SIRT7 in HPAEC also resulted in activation of the Rho signaling pathway involved in endothelial barrier disruption with increased levels of phosphorylated myosin light chain 2 (MLC2) under basal conditions and in response to thrombin stimulation (Supplementary Figure 12), suggesting an additional molecular mechanism for increased barrier permeability induced by SIRT7 suppression in endothelial cell cultures.

## Discussion

To our knowledge, this is the first report on the role of SIRT7, a member of the longevity-associated SIRT family, in LPS-induced ALI. Cellular and molecular mechanisms of aging have recently begun to be recognized as contributing factors to the development of vascular dysfunction and ALI^9,10,48^. We found significantly lower SIRT7 mRNA levels in lung tissues from aged compared to young mice and in murine lungs challenged with LPS or bleomycin. Conversely, acute LPS or bleomycin challenge induced pro-inflammatory and pro-fibrotic responses with increases in ICAM1, VCAM, IL6, and collagen chains in murine lung. Our findings are in agreement with studies showing suppressed SIRT1 and SIRT3 levels in mouse lung tissues after LPS exposure^42–44^ and suggest a protective role of SIRT7 in mitigating inflammatory and fibrotic responses characteristic of ALI.

To examine cell-specific effects of SIRT7 loss in ALI, we focused our next experiments on acute inflammatory responses in cultured primary pulmonary endothelial cells. Contrary to the association between suppressed SIRT7 levels and increased inflammatory markers seen in LPS-challenged murine lung tissues, SIRT7 silencing in HPAEC or HPMVEC significantly lowered LPS-induced increases in ICAM1, VCAM, IL6, and IL8 mRNA or protein levels. Similar effects were observed in endothelial cells isolated from *Sirt7*-deficient mice. These findings were accompanied by decreased total NFκB levels in SIRT7-silenced HPMVEC and decreased NFκB phosphorylation and nuclear translocation in SIRT7-silenced HPAEC. Although these findings suggest a direct effect of SIRT7 silencing on NFκB activity in the endothelium, the effect is modest, and other, additional mechanisms are likely responsible for the significant anti-inflammatory response observed in SIRT7-silenced endothelial cell cultures. SIRT7 silencing induced EndoMT in endothelial cell cultures with loss of the endothelial cell adhesion proteins VE-Cadherin and PECAM1 and increases in αSMA and the transcription factor Snail. SIRT7-silenced HPAEC cultures simultaneously expressed significantly more COL1A2 and less IL6, IL8, ICAM1, and VCAM1 mRNA levels at multiple time points after LPS stimulation, suggesting the anti-inflammatory response to SIRT7 depletion may be due to the development of a mesenchymal phenotype. Furthermore, SIRT7-silenced endothelial cultures produced higher levels of TGFβ1, a potent anti-inflammatory cytokine that is released during the fibroproliferative stage of ALI and may contribute both to EndoMT^49,50^ and blunted inflammatory responses to LPS in the lung endothelium.

TGFβ1 has also been shown by our group and others to activate the Rho pathway and induce endothelial barrier dysfunction^51,52^ and could explain the increases in vascular permeability, F-actin stress fibers, and Rho signaling we observed with SIRT7-silencing in pulmonary endothelial cells. β-Catenin, a component of the VE-Cadherin cell adhesion complex, is another important regulator of endothelial barrier integrity. β-Catenin loss has been demonstrated following endothelial barrier-disruptive insults^53^, and restoration of β-Catenin is induced by compounds that protect against LPS-induced ALI, in part by restoring depleted SIRT3 levels^54^. SIRT7 suppression increased phosphorylated levels of β-Catenin relative to total levels at a residue that targets β-Catenin for degradation by the ubiquitin proteasomal pathway, suggesting that SIRT7 loss may contribute to vascular dysfunction, at least in part, by targeting β-Catenin for degradation in the cytoplasm. Together, these findings show that SIRT7 silencing disrupts endothelial cellular junctions at baseline and in response to barrier-disruptive agonists, and are consistent with the barrier-protective roles of other sirtuins (SIRT1 and SIRT3) in ALI^42,46,47,54^. Although studies on vascular permeability were conducted in HPAEC, SIRT7 silencing in both HPAEC and HPMVEC resulted in loss of endothelial cellular adhesion proteins, which may explain increased capillary permeability induced by SIRT7 loss in vivo.

Although Rho activation and increased endothelial barrier permeability is typically accompanied by increased inflammation in response to pro-inflammatory agonists^55^, this relationship may no longer apply to endothelial cells that have acquired a mesenchymal phenotype such as that induced by SIRT7 depletion. Together, our findings suggest that EndoMT is a central mechanism both for the anti-inflammatory effects and increased vascular permeability induced by SIRT7 depletion in pulmonary endothelium. Few studies have examined EndoMT specifically mediated by SIRTs and, to our knowledge, this is the first to show that SIRT7 loss induces EndoMT in the lung vasculature. SIRT1 loss has been shown to induce EndoMT and upregulate TGFβ levels in human umbilical vein endothelial cells^56^, and SIRT3 KO mice undergo EndoMT in renal fibrosis^57^, both of which support our findings. Several other pathways induced by molecules such as Wnt, Notch1, and Sonic Hh have been described in EndoMT^50^. Their potential regulation by SIRT7 will be addressed in future studies.

It may seem paradoxical that SIRT7 loss is associated with increased inflammation in lung tissues in vivo, but suppresses LPS-induced pro-inflammatory responses in endothelial cell cultures. However, the function of SIRTs varies depending on cellular context, and other cell types, such as immune or epithelial cells, may be contributing to increased inflammatory responses in the lung mediated by LPS-induced SIRT7 loss. Although we did not observe a significant effect of LPS on SIRT7 levels in pulmonary endothelial cells, LPS stimulation significantly decreased SIRT7 levels in primary small airway epithelial cells. Furthermore, silencing SIRT7 in lung epithelium had a pro-inflammatory effect. Suppressed cytokine release in response to LPS occurring with SIRT7 loss may be specific to endothelial cells and interfere with reparative immune responses such as inflammatory cell recruitment, elimination of infectious pathogens, and tissue repair. Alternatively, SIRT7 loss in the endothelium may be a compensatory response to limit LPS-induced pro-inflammatory damage to the lung.

In summary, we have found that loss of SIRT7, a member of the family of sirtuin youth genes, occurs during LPS- or bleomycin-induced ALI and induces a mesenchymal phenotype in pulmonary endothelial cells, with impaired inflammatory responses to LPS, increased vascular permeability, and loss of cell junction proteins. SIRT7 loss associated with aging or induced by LPS exposure has an opposite, pro-inflammatory effect in airway epithelium. We propose a model where LPS- or aging-induced SIRT7 deficiency promotes ALI by inducing inflammation in the lung epithelium and promoting EndoMT in the lung endothelium. Although EndoMT suppresses inflammatory responses in pulmonary endothelial cells, it may indirectly contribute to increased inflammation in lung tissues by disrupting endothelial cell junctions and increasing vascular barrier permeability. SIRT7 loss in pulmonary endothelial cells may increase susceptibility to acute lung injury and impair its resolution or be a compensatory response to attenuate endothelial pro-inflammatory responses in lung injury. Better understanding of mechanisms occurring with SIRT7 loss during aging should improve efforts at understanding and treating vascular dysfunction in ALI.

## Methods

### Cell culture

Primary adult human pulmonary artery endothelial cells (HPAEC) and pulmonary microvascular endothelial cells (HPMVEC) were purchased from Lonza (Walkersville, MD) or PromoCell (Heidelberg, Germany) and cultured in endothelial basal medium supplemented with growth factors and 10% fetal bovine serum (FBS) from Gibco (Gaithersburg, MD). Endothelial cell cultures were maintained in T75 culture flasks in a humidified atmosphere with 5% CO_2_ at 37 °C. For experiments, cell cultures were incubated with the same endothelial growth media containing 2% FBS for 15 min to two hours prior to testing. Cells were passaged by washing with PBS, trypsinizing with 3 ml 0.25% trypsin–EDTA (Gibco), and reconstituting cells in growth media with 10% FBS before transfer to new T75 flasks, dishes, or 6- or 12-well plates. Experiments were performed with endothelial cell culture passages four to nine. Additional experiments were performed in primary human small airway epithelial cells, which were purchased from Lonza and cultured in small airway epithelial cell basal medium supplemented with serum and growth factors.

### In vivo experiments

Lung tissues from 5 week-old and 18 month-old wild-type C57BL/6 mice were used to evaluate the effects of age on SIRT7 expression. To evaluate the effects of LPS in vivo, 10 week-old adult male C57BL/6 J or A/J mice obtained from Jackson Laboratory (Bar Harbor, ME) were administered 100 µg of LPS from Pseudomonas aeruginosa (Millipore Sigma, St. Louis, MO) dissolved in 50 µL of PBS. LPS or PBS alone were administered intranasally to anesthetized mice, which were euthanized 24 h later. To model pulmonary inflammation and fibrosis in vivo, 10–12 week-old wild-type female C57BL/6 mice were treated with intratracheal instillations of bleomycin or sterile PBS and lung tissues collected for analysis 14 days later as previously described^36^. Endothelial cells were derived from 10–12 week-old male wild-type and *Sirt7* deficient mice, a kind gift of Dr. Lourdes Serrano, Rutgers University^58^. Heterozygous mice (*Sirt7*^+*/−*^) were used for experiments due to difficulty breeding homozygous (*Sirt7*^*−/−*^) mice and observed twofold decreases in SIRT7 expression in heterozygous animals. All animal experiments were performed with approval from the SUNY Downstate Medical Center and University of Maryland Institutional Animal Care and Use Committees in accordance with relevant guidelines and regulations.

### Isolation, transfection, and stimulation of primary endothelial cell cultures

To deplete SIRT7 levels, HPAEC or HPMVEC were transiently transfected with human SIRT7 or scrambled control (CTRL) siRNA (Qiagen, Valencia, CA). Endothelial cells were seeded in D60 dishes, 6-well or 12-well plates, glass coverslips (for IF or XPerT assays), or microelectrodes (for ECIS experiments) and transfections performed on adherent cells at 60–80% confluency the following day using Lipofectamine RNAiMAX Transfection Reagent (Invitrogen, Thermo Fisher Scientific, Waltham, MA) according to the manufacturer’s instructions. In some experiments, transfections were performed in suspension cultures by electroporation using the Amaxa 4D nucleofector, P5 nucleofector solution, and supplement from Lonza. For each transfection, 0.5–0.7 million cells were electroporated with 30 pmol of siRNA using program CA-167 as described in the basic protocol for primary mammalian endothelial cells (Lonza) and transferred to six- or 12-well plates. New growth media with 10% FBS was added the next day, and experiments performed 48 h after transfection. SIRT7 depletion was confirmed by Western blotting (WB) or RT-qPCR for all transfections with SIRT7 siRNA.

Endothelial cells were derived from wild-type and *Sirt7* heterozygous mice as previously described^59^. Mouse lungs were harvested, minced with scissors, and digested with Type I collagenase (Thermo Fisher Scientific). The cell suspension was incubated with sheep anti-Rat IgG dynabeads (Thermo Fisher Scientific) conjugated to rat anti-mouse CD31 antibody (BD Bioscience) for 20 min and seeded in a 6-well plate. When confluent, cells were trypsinized, incubated with dynabeads conjugated to rat anti-mouse ICAM2 (BD Bioscience) for 20 min, and seeded in new 6-well plates. Cells were stimulated with LPS when confluent.

LPS used for cell cultures was purchased from Santa Cruz Biotechnology (Dallas, TX) and dissolved in water to 1 mg/ml stock concentration. Endothelial cell cultures were incubated in growth medium with 2% FBS for 15–30 min prior to stimulation with LPS at 50 ng/ml or 100 ng/ml concentrations and cell lysates collected 6–48 h later for analyses. Small airway epithelial cells were stimulated with 1 ug/ml LPS for 24 h. Thrombin from human plasma was purchased from Millipore Sigma and dissolved in water to 10 U/ml concentration. Endothelial cells were incubated in growth medium with 2% FBS for two hours prior to stimulation with thrombin at 0.1–0.2 U/ml and cell lysates collected 15 min later for analyses.

### RNA isolation, cDNA synthesis, and real-time PCR

Total RNA was isolated from endothelial cells or homogenized mouse lung tissue using TRIzol reagent (Ambion, Life Technologies, Carlsbad, CA). RNA was isolated by phase separation with 0.2 ml chloroform per 1 ml TRIzol followed by precipitation overnight at − 20 °C with isopropanol. After washing with ethanol, RNA was dissolved in PCR-certified water and RNA concentration and purity were determined using the NanoDrop One Spectrophotometer (Thermo Fisher Scientific). In some experiments, RNA was isolated using the RNeasy mini kit (Qiagen) as described in the protocol. Complementary cDNA was synthesized from 1–2 ug of RNA using the iScript cDNA synthesis kit from Bio-Rad (Hercules, CA) according to the manufacturer’s protocol. RT-qPCR was performed with 5 ul PerfeCTa SYBR Green FastMix (Quantabio, Beverly, MA), 3 ul of PCR-certified water, 1 ul of primer, and 1 ul of cDNA per reaction. Mouse *18 s*, *Sirt7*, *Icam1*, *Vcam1*, *Col1a1*, *Col1a2*, and *Col3a1* and human 18S, SIRT7, COL1A1, COL1A2, COL3A1, α-SMA, SMAD2, SMAD3, TGFβ1, TGFβ-R1, and TGFβ-R2 primers were obtained from Qiagen. All other primers, which were designed and validated in-house, are listed in Table 1.

**Table 1:** Primers used for quantitative RT-qPCR.

Gene	Primer sequences^a^
Human SIRT7	ACGCCAAATACTTGGTCGTCT
	AGCACTAACGCTTCTCCCTTT
Human ICAM1	TTGGGCATAGAGACCCCGTT
	GCACATTGCTCAGTTCATACACC
Human VCAM1	CAGTAAGGCAGGCTGTAAAAGA
	TGGAGCTGGTAGACCCTCG
Human IL6	CCTGAACCTTCCAAAGATGGC
	TTCACCAGGCAAGTCTCCTCA
Human IL8	TGACTTCCAAGCTGGCCGTGG
	ACTGCACCTTCACACAGAGCTGC
Human GAPDH	ATGGGGAAGGTGAAGGTC
	GGGGTCATTGATGGCAACAATA
Mouse *Sirt7*	ACCACTGCCTTACCTCACTC
	CTGTGCCGCATACCCAATAC
Mouse *Icam1*	CTGCCTCTGAAGCTCGGATA
	GTCACCTCTACCAAGGCAGT
Mouse *Vcam1*	ACGAGGCTGGAATTAGCAGA
	TCGGGCACATTTCCACAAG
Mouse *IL6*	CCGGAGAGGAGACTTCACAG
	TCCACGATTTCCCAGAGAAC
Mouse *β-actin*	GTTGGAGCAAACATCCCCCA
	CGCGACCATCCTCCTCTTAG
Mouse *Gapdh*	AATGTGTCCGTCGTGGATCT
	AGACAACCTGGTCCTCAGTG

^a^For each primer set, top sequence denotes forward primer and bottom sequence reverse primer

### Immunoblotting and immunofluorescence

For Western blot analyses, endothelial cell cultures were washed with ice-cold PBS and lysed with Laemmli sample buffer. Samples were reduced and denatured by boiling for 5 min, and electrophoresis performed with 8–15% tris–glycine polyacrylamide gels. Protein bands were transferred to PVDF membranes (Thermo Fisher Scientific). Membranes were blocked at room temperature for 1–2 h and incubated overnight at 4 °C with primary antibodies dissolved in Tris buffered saline (TBS, Quality Biological) with 3% BSA (Sigma) and 0.1% Tween 20 (Thermo Fisher Scientific). After washing with TBS and 0.1% Tween-20, membranes were incubated with secondary HRP-linked anti-rabbit or anti-mouse antibodies (Santa Cruz Biotechnology) at 1:4,000 dilution for 1 h at room temperature. Membrane protein bands were developed with SuperSignal West Pico Chemiluminescent or Pierce ECL substrate (Thermo Fisher Scientific) and HyBlot CL Audiography film (Thomas Scientific, Swedesboro, NJ). Gel images were scanned using LabScan software (GE Healthcare) and saved as TIFF files at 600 dpi resolution. In all WB panels, white spaces demarcate different proteins probed with different primary antibodies for the same group of samples, either from the same PVDF membrane or from a different gel and membrane.

For immunofluorescence (IF) experiments, HPAEC or HPMVEC were seeded on glass coverslips, transfected with CTRL or SIRT7 siRNA, and fixed in 3.7% formaldehyde for 10 min at room temperature 48 h after transfection. Cells were washed with PBS, permeabilized with 0.1% Triton X-100 (Sigma) in PBS for 15 min, blocked with 2% BSA and 0.1% Triton X-100 in PBS for 30 min, and incubated overnight at 4 °C with 1:1,000 anti-rabbit VE-Cadherin antibody (Cayman Chemical, Ann Arbor, MI) or 1:150 anti-mouse PECAM1 antibody (Cell Signaling Technology, Danvers, MA). After washing, cells were incubated with 1:500 Alexa Fluor 488-conjugated anti-rabbit or anti-mouse IgG antibody or Alexa Fluor 594-conjugated Texas Red-X Phalloidin (Thermo Fisher Scientific) for 1 h at room temperature. All images were captured using the EVOS FL Auto 2 Cell Imaging System (Thermo Fisher Scientific). Protein densities (sum of pixel values) for WB and IF experiments were measured with ImageJ^60^.

### ELISA

Media from HPAEC or HPMVEC transfected with CTRL or SIRT7 siRNA was collected 6–8 h after stimulation with LPS, centrifuged, and transferred to new tubes. SIRT7 protein depletion and equal total protein levels were confirmed by Western blotting with SIRT7 and α-tubulin primary antibodies, respectively. Soluble ICAM1, IL6, and IL8 levels in collected media samples were determined using DuoSet solid phase sandwich ELISA kits from R&D Systems (Minneapolis, MN) according to the manufacturer’s protocol.

### Isolation of cytoplasmic and nuclear cell lysates

HPAEC were seeded in D60 dishes, cell cultures transfected with CTRL or SIRT7 siRNA, and cytoplasmic and nuclear cell fractions isolated 48 h later using the nuclear extract kit from Active Motif (Carlsbad, CA) according to the manufacturer’s instructions.

### Measurements of endothelial monolayer permeability

Cellular barrier properties were analyzed by measurements of transendothelial electrical resistance (TER) across confluent human pulmonary artery endothelial monolayers using an electrical cell-substrate impedance sensing system (Applied BioPhysics, Troy, NY, USA) as previously described^61^. Cells were cultured on microelectrodes in growth media with 10% FBS. Media was changed to growth media containing 2% FBS and a 4,000-Hz AC signal with 1-V amplitude applied to the cells. Cells were equilibrated until the electrical resistance achieved a steady state prior to the addition of LPS or thrombin, and analyses performed 24 h later.

Permeability of endothelial cell monolayers for macromolecules was assessed using the Express Micromolecule Permeability Testing Assay (XPerT) as previously described^62,63^. HPAEC were seeded on glass coverslips coated with EZ-Link Sulfo-NHS-SS-Biotin (Thermo Fisher Scientific) dissolved in gelatin (Sigma) and, 48 h after transfection with CTRL or SIRT7 siRNA and stimulation with LPS for 6 h, FITC-avidin tracer (Thermo Fisher Scientific) at 1:200 dilution added to the culture media for 2 min. Unbound FITC-avidin was removed by washing with pre-warmed (37 °C) PBS and cells fixed with 3.7% formaldehyde in PBS for 10 min. Cells were washed with PBS, mounted on glass slides, and images taken using the EVOS FL Auto 2 Cell Imaging System (Thermo Fisher Scientific). Fluorescence was measured using Image J^60^.

### Antibodies and reagents

Primary rabbit antibodies to SIRT7, VCAM1, phosphorylated (Ser536) and total NF-κB p65, phosphorylated (Thr18/Ser19) and total Myosin Light Chain 2 (MLC2), Snail, HDAC2, phosphorylated (Ser33/37/Thr41) β-Catenin and mouse antibody to PECAM1 were purchased from Cell Signaling Technology. Rabbit α-SMA was purchased from Abcam (Cambridge, MA); rabbit total β-Catenin from GenTex (Irvine, CA); rabbit VE-Cadherin from Cayman Chemical; mouse ICAM1 from Santa Cruz Biotechnology; and α-tubulin from Millipore Sigma.

### Statistical analyses

Data were analyzed using GraphPad Prism 8 software and are reported as mean values +/− SD. Differences between two groups were assessed using the Student’s two-tailed unequal variance *t* test or the Mann–Whitney *U*-test, as indicated for specific experiments. *P* values less than 0.05 were considered statistically significant.

## Supplementary information

Supplementary Information.
