# Supplementary material for: SIRT7 deficiency suppresses inflammation, induces EndoMT, and increases vascular permeability in primary pulmonary endothelial cells
Source: Sci Rep. 2020 Jul 27;10:12497. doi: 10.1038/s41598-020-69236-z (PMC7385158; doi:10.1038/s41598-020-69236-z)

# SIRT7 Deficiency Suppresses Inflammation, Induces EndoMT, and Increases Vascular Permeability in Primary Pulmonary Endothelial Cells

Anne E. Wyman[1,2,3,4*], Trang T.T. Nguyen[2], Pratap Karki[2], Mohan E. Tulapurkar[2], Chen-Ou Zhang[2], Junghyun Kim[2], Theresa G. Feng[5], Abdoulaye J. Dabo[4], Nevins W. Todd[2,3], Irina G. Luzina[2,3], Patrick Geraghty[4], Robert F. Foronjy[4], Jeffrey D. Hasday[2,3], Anna A. Birukova[2], Sergei P. Atamas[2,3], and Konstantin G. Birukov[2]

[1]Geriatric Research Education and Clinical Center (GRECC), VA Maryland Health Care Center, Baltimore VA Medical Center, [2]Department of Medicine, University of Maryland School of Medicine, [3]Research Service, Baltimore VA Medical Center, [4]Departments of Medicine and Cell Biology, SUNY Downstate Health Sciences University, [5]Department of Anesthesiology, SUNY Downstate Health Sciences University

[*]Address for correspondence: Anne E. Wyman, MD. SUNY Downstate Health Sciences University, 450 Clarkson Ave, MSC:19, Brooklyn, NY 11203. Anne.Wyman@downstate.edu.

Supplementary Figure 1

Full-length WBs for SIRT7 (**A**), ICAM1 (**B**), α-tubulin (**C**), VCAM1 (**D**), and α-tubulin (**E**) for panel C in Figure 3. α-tubulin in panel **C** is from the same WB membrane as ICAM1; α-tubulin in panel **E** is from the same membrane as VCAM1.

Supplementary Figure 2

Full-length WBs for SIRT7 (**A**), VCAM1 (**B**), NFκB (**C**) and α-tubulin (**D**) for panel D in Figure 3.

Supplementary Figure 3

Effects of LPS on SIRT7 and SIRT7 silencing on inflammatory mediators in airway epithelial cells. **A:** SIRT7 mRNA normalized to β-actin levels in airway epithelial cells stimulated with PBS (□, n=5) or 1 μg/ml LPS (■, n=3) for 24 hours. **B:** SIRT7, IL6, and IL8 mRNA levels normalized to β-actin in airway epithelial cells transfected with CTRL (□, n=3) or SIRT7 (■, n=3) siRNA. Data are expressed as fold changes relative to average mRNA levels in unstimulated (LPS 0) cell cultures (**A**) or CTRL-silenced cell cultures (**B**). Bars represent averages and brackets standard deviations. P-values were calculated using the Student's *t*-test.

Supplementary Figure 4

Full-length WBs for SIRT7 (**A**), NFκB (**B**), α-tubulin (**C**), and HDAC2 (**D**) for panel A in Figure 4.

Supplementary Figure 5

Full-length WBs for SIRT7 (**A**), phosphorylated NFκB (**B**), and α-tubulin (**C**) for panel B in Figure 4.

Supplementary Figure 6

Phase contrast images of CTRL- and SIRT7-silenced HPAEC cultures viewed under the light microscope at 10x objective 48 hours after transfection.

Supplementary Figure 7

Time course of LPS effects on pro-inflammatory and pro-fibrotic gene expression in CTRL- and SIRT7-silenced HPAEC. mRNA levels of each indicated RT-qPCR target

at each time point in CTRL-silenced (□) or SIRT7-silenced (■) HPAEC cultures were normalized to GAPDH and expressed as fold changes relative to average mRNA levels in unstimulated (LPS 0), CTRL-silenced cell cultures. Bars represent averages and brackets standard deviations of measurements for two samples per condition. P-values were calculated using the Student's *t*-test. Significant differences ($P < 0.05$) between and CTRL- and SIRT7-silenced cultures for each LPS concentration are denoted by stars.

## Supplementary Figure 8

Full-length WBs for SIRT7 (**A**), VE-Cadherin (**B**), GAPDH (**C**), PECAM1 (**D**), α-tubulin (**E**), α-SMA (**F**), SNAIL (**G**), and GAPDH (**H**) for panel A in Figure 6. Samples for SIRT7 (**A**), VE-Cadherin (**B**), and GAPDH (**C**) were run on a 15-well gel. These images were scaled up proportionally to match widths of protein bands in panels **D**-**H**, which represent the same samples run on a 10-well gel.

## Supplementary Figure 9

Full-length WBs for SIRT7 (**A**), PECAM1 (**B**), and α-tubulin (**C**) for panel C in Figure 6.

## Supplementary Figure 10

HPAEC plated on microelectrodes were transfected with CTRL or SIRT7 siRNA and transendothelial electric resistance (TER) measurements performed after stimulation with LPS (100 ng/ml) (**A**) or thrombin (0.2 U/ml) (**B**). Resistance data were normalized to the initial voltage and plotted as a normalized resistance. Data points represent averages and brackets standard deviations of three samples per condition in (**A**) and two samples per condition in (**B**). Experiments were performed on at least two separate occasions with similar results.

## Supplementary Figure 11

Full-length WBs for SIRT7 (**A**), total β-Catenin (**B**), α-tubulin (**C**), phosphorylated β-Catenin (**D**), and α-tubulin (**E**) corresponding to panel C in Figure 7.

Supplementary Figure 12

Effects of SIRT7 silencing on Rho signaling in HPAEC. **A:** Effect of SIRT7 silencing in HPAEC on thrombin-induced protein levels of phosphorylated and total myosin light chain 2 after thrombin stimulation for 15 minutes. Densitometry measurements relative to α-tubulin (RDU) are shown. Full-length WBs for SIRT7 (**B**), phosphorylated MLC2 (**C**), total MLC2 (**D**), and α-tubulin (**E**) in panel A.

## Supplementary Figure 1

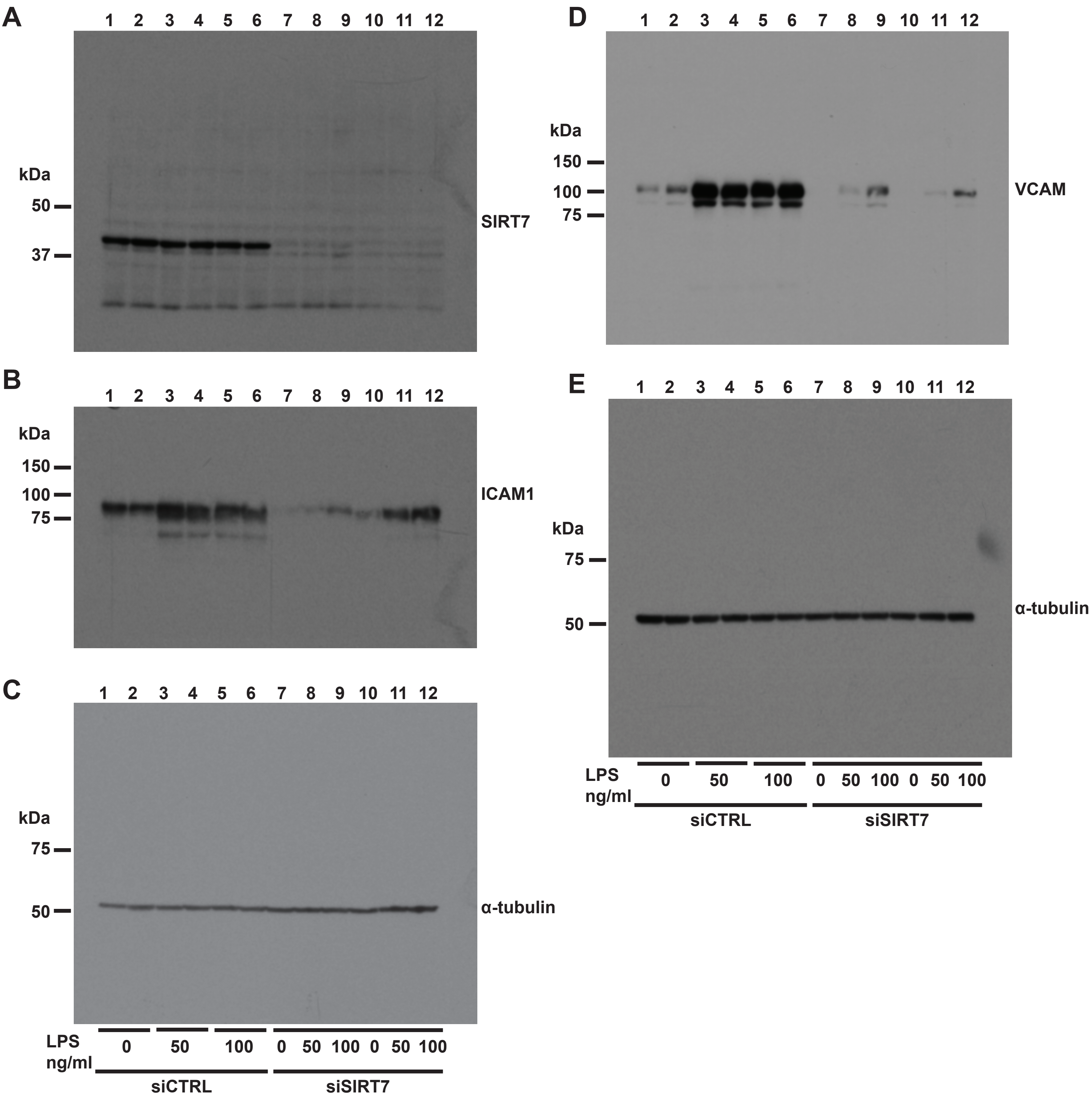

**Supplementary Figure 2**

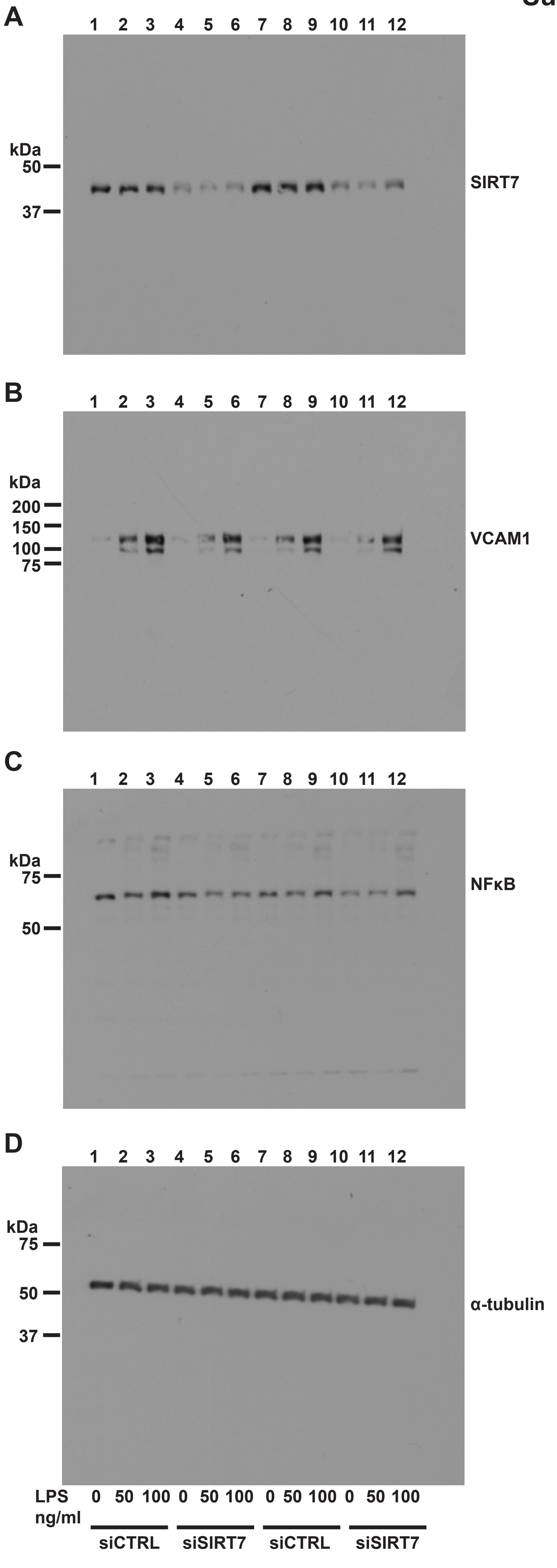

## Supplementary Figure 3

A

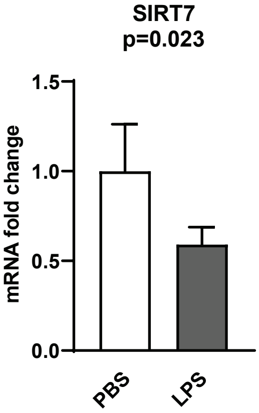


B

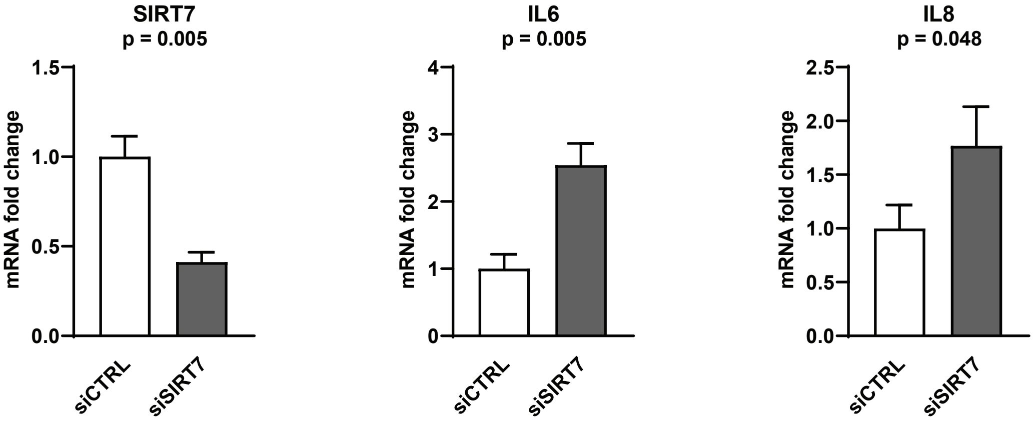

**Supplementary Figure 4**

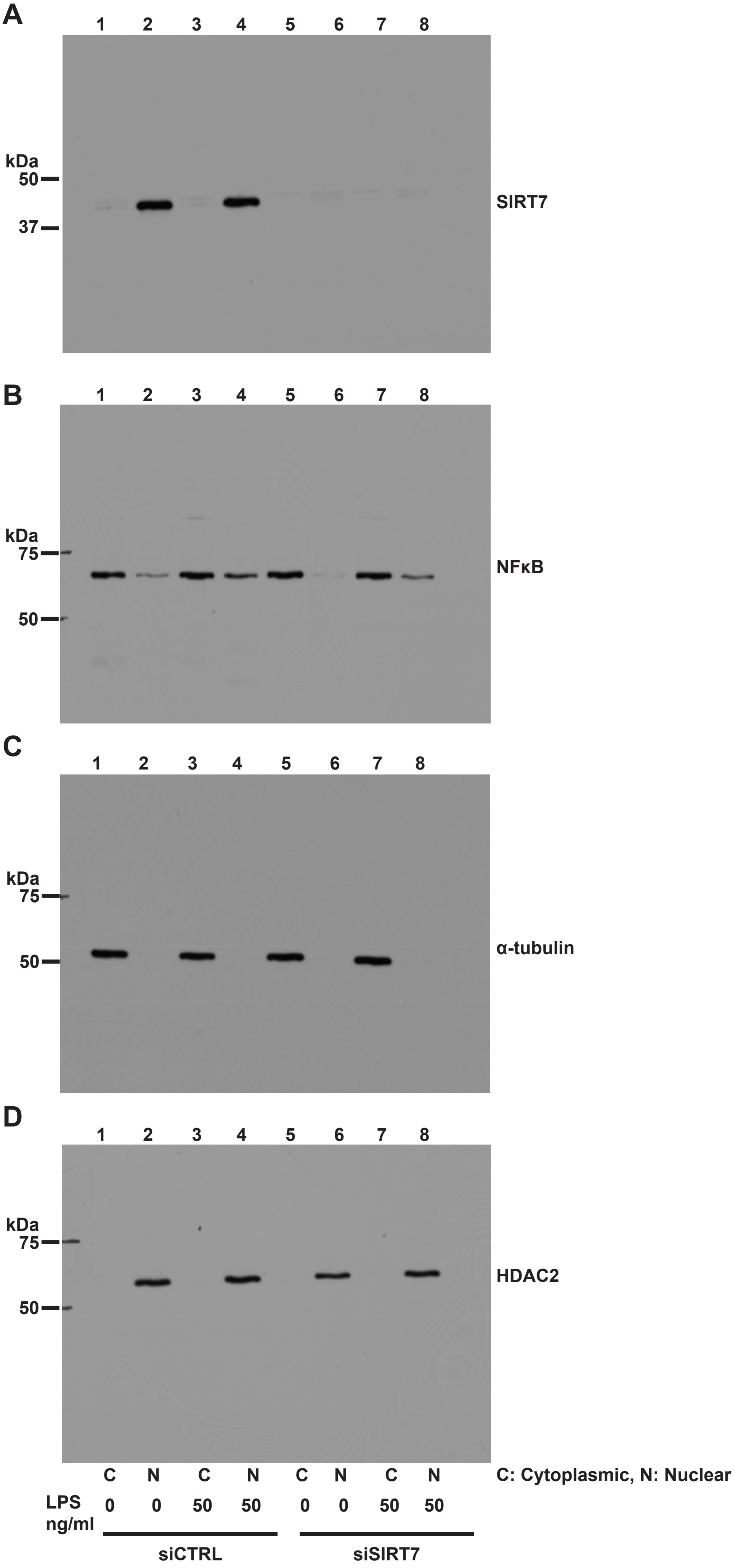

## Supplementary Figure 5

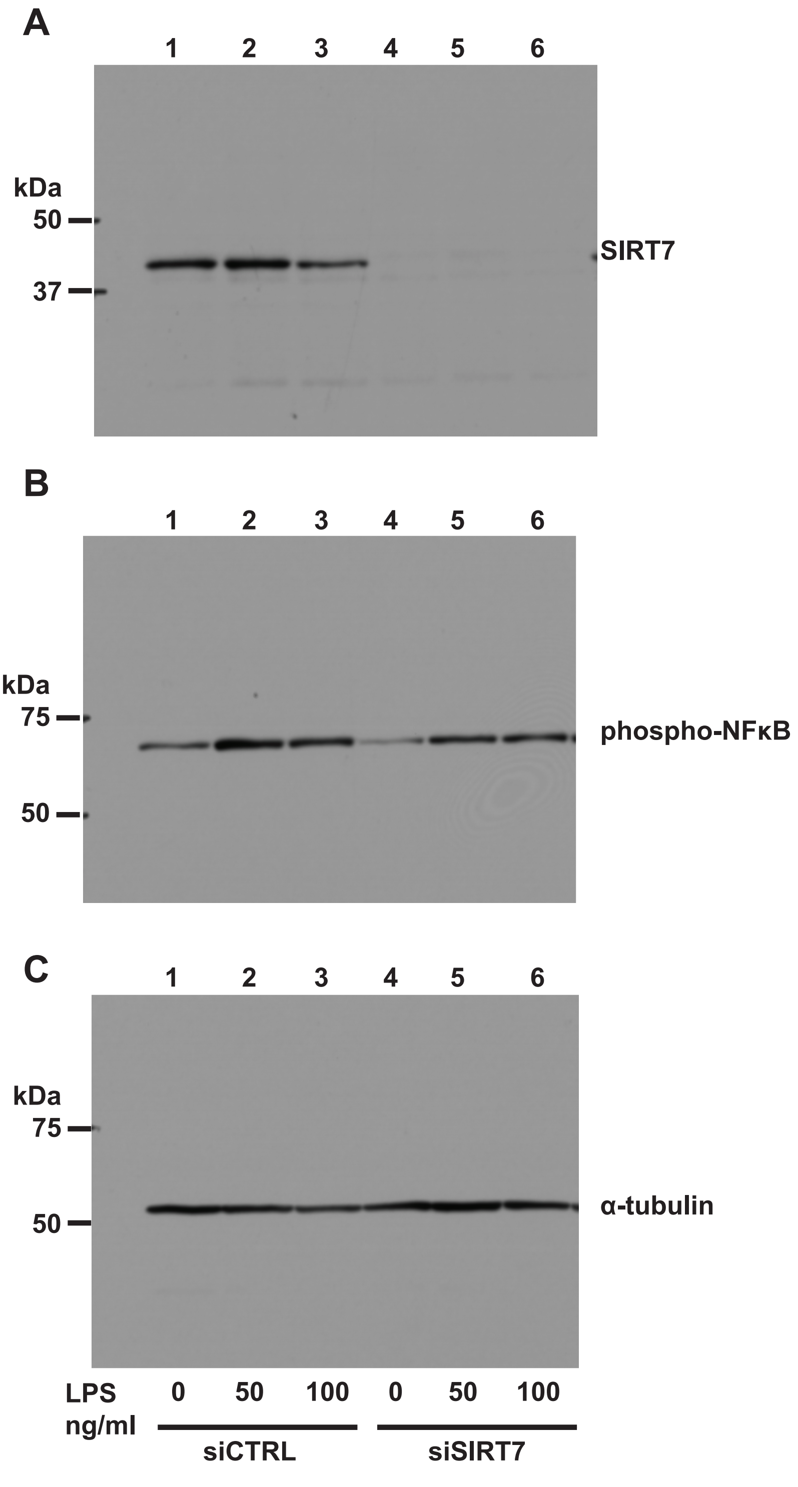

**Supplementary Figure 6**

**siCTRL**

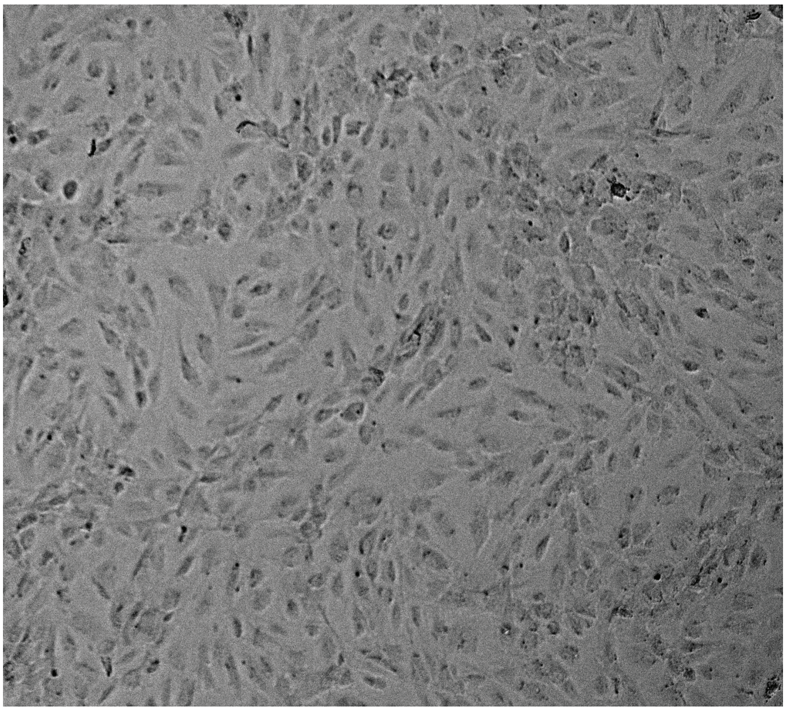

**siSIRT7**

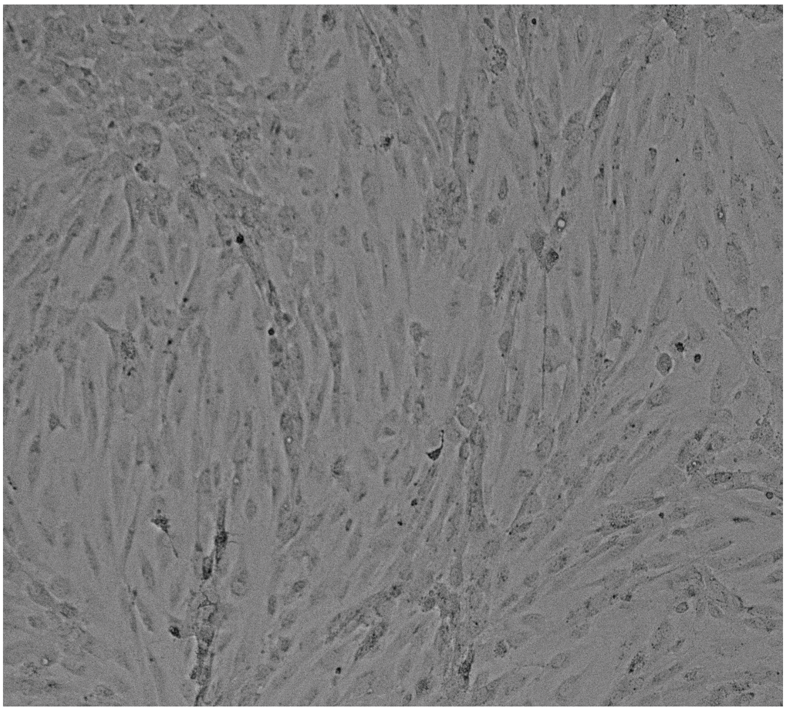

# Supplementary Figure 7

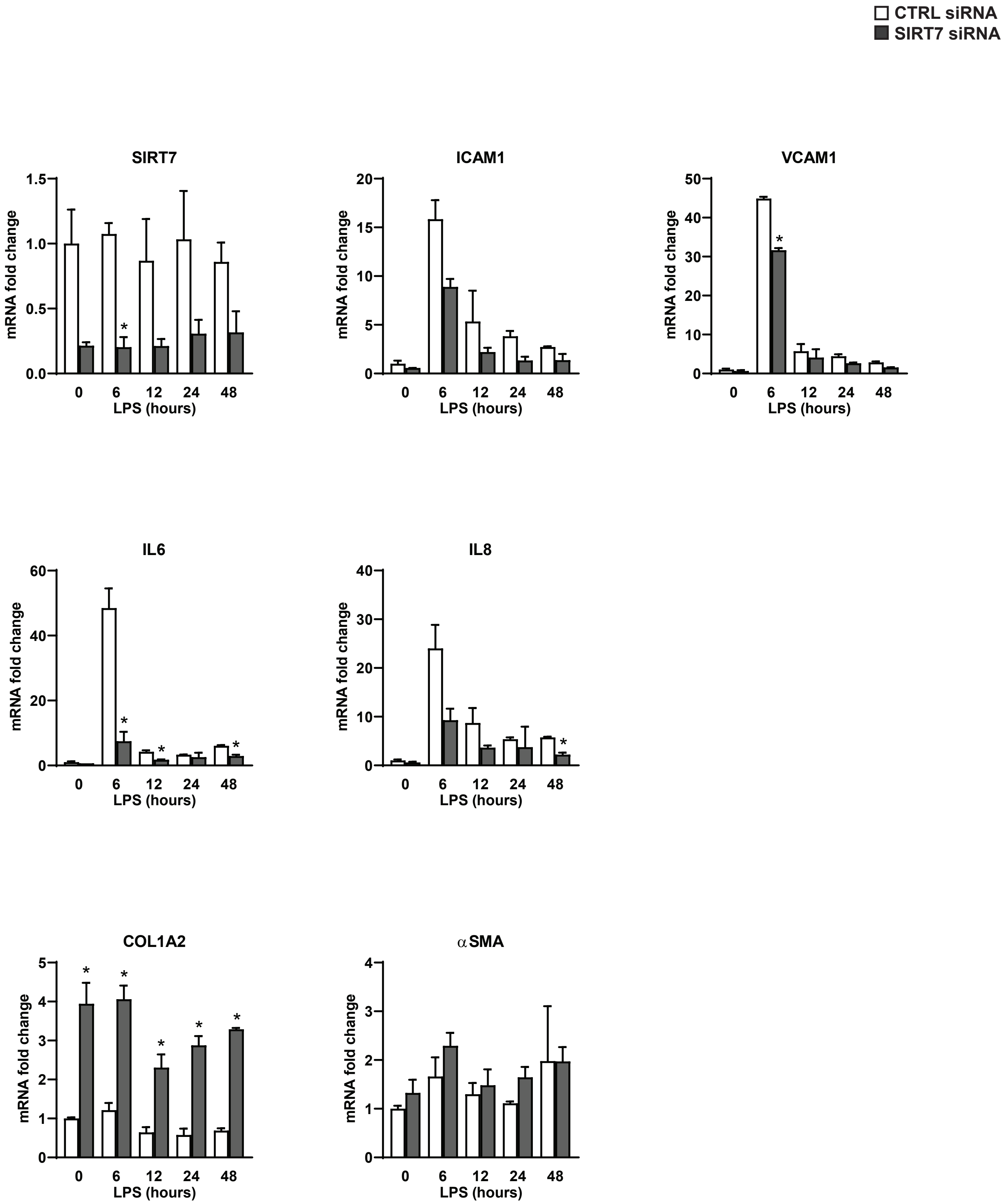

**Supplementary Figure 8**

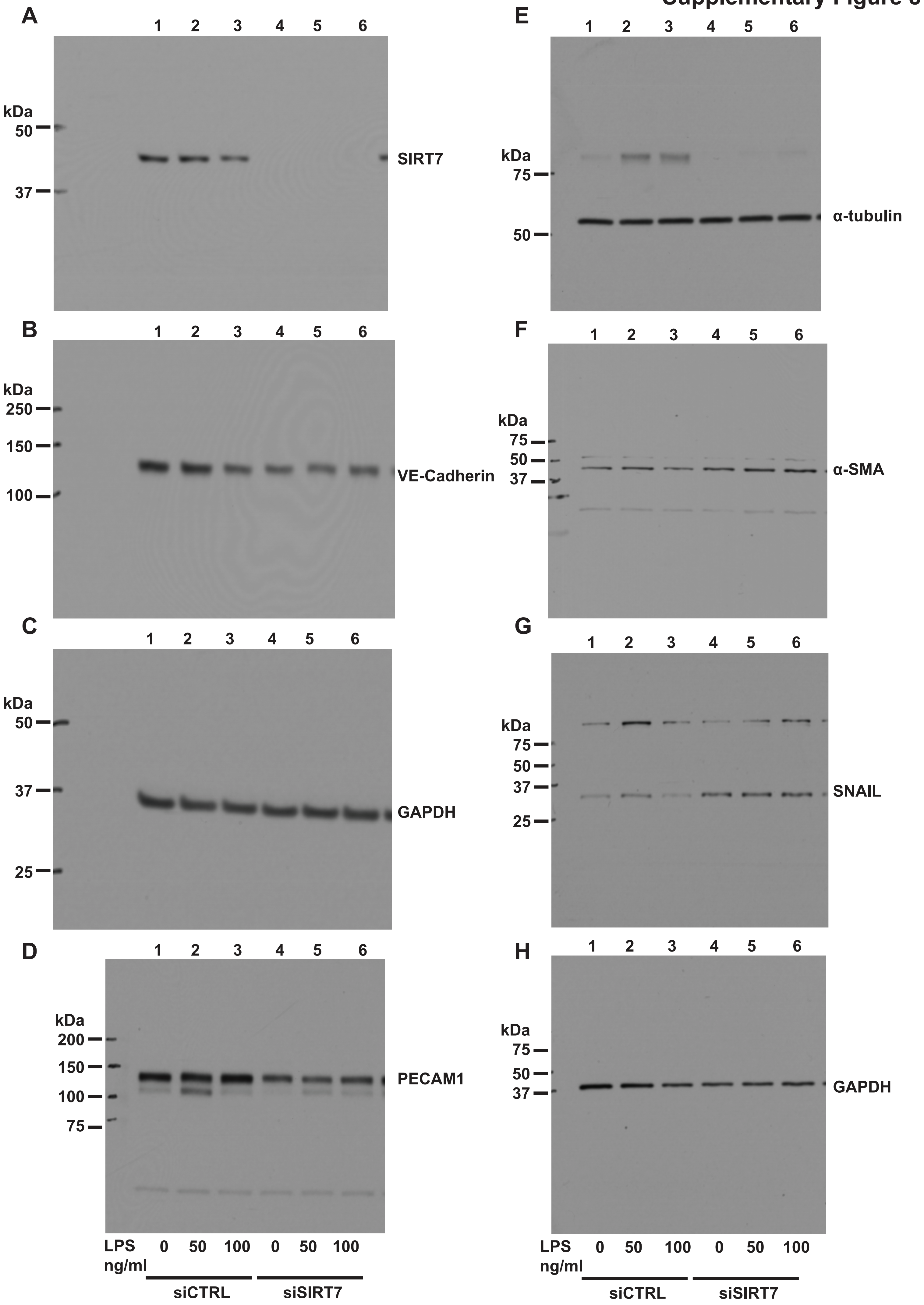

**Supplementary Figure 9**

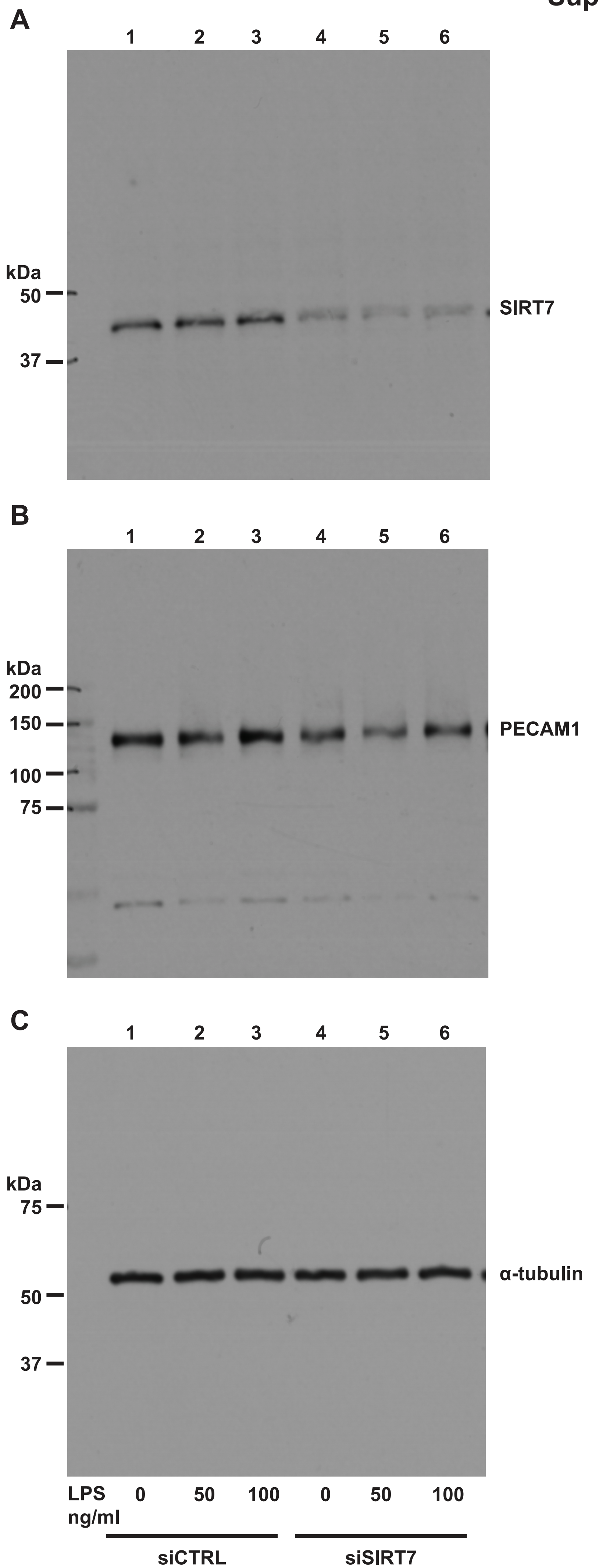

**A**

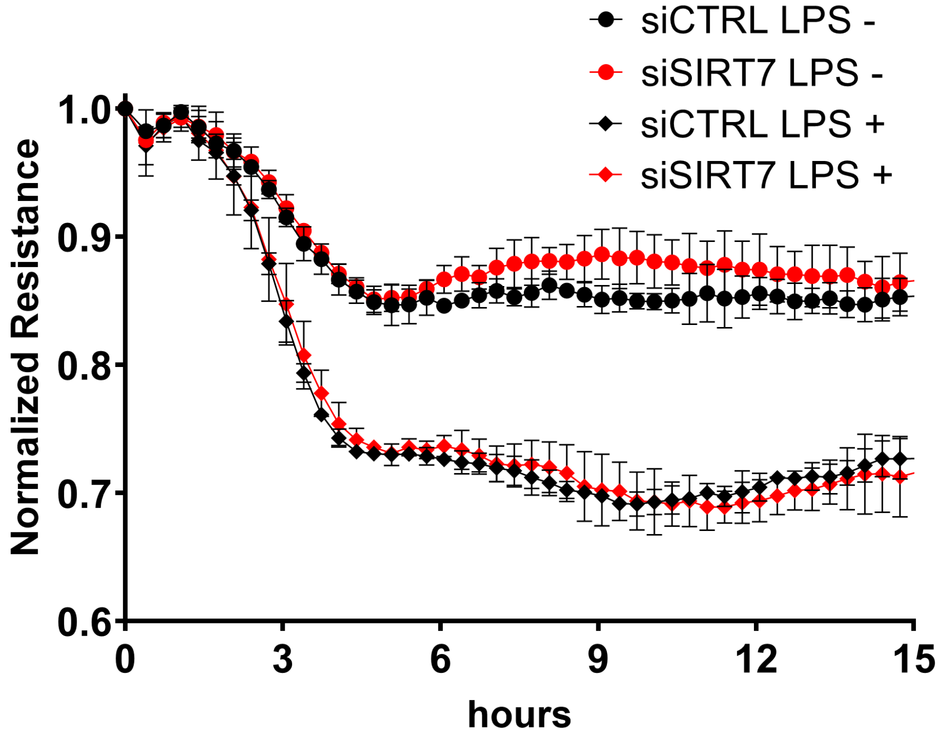
siCTRL LPS -
siSIRT7 LPS -
siCTRL LPS +
siSIRT7 LPS +
Normalized Resistance
1.0
0.9
0.8
0.7
0.6
0
3
6
9
12
15
hours

**B**

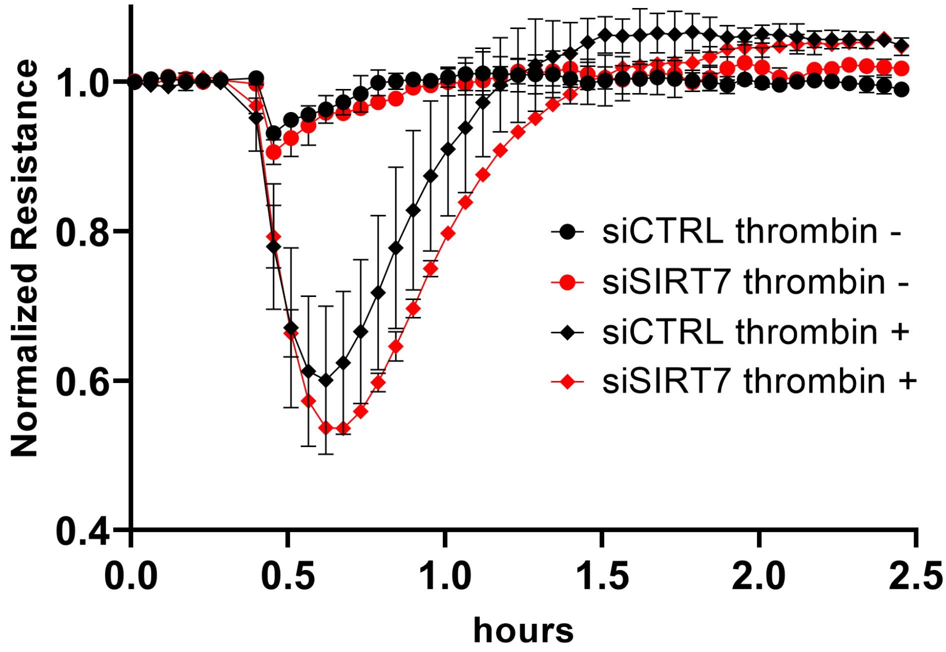
siCTRL thrombin -
siSIRT7 thrombin -
siCTRL thrombin +
siSIRT7 thrombin +
Normalized Resistance
1.0
0.8
0.6
0.4
0.0
0.5
1.0
1.5
2.0
2.5
hours

## Supplementary Figure 11

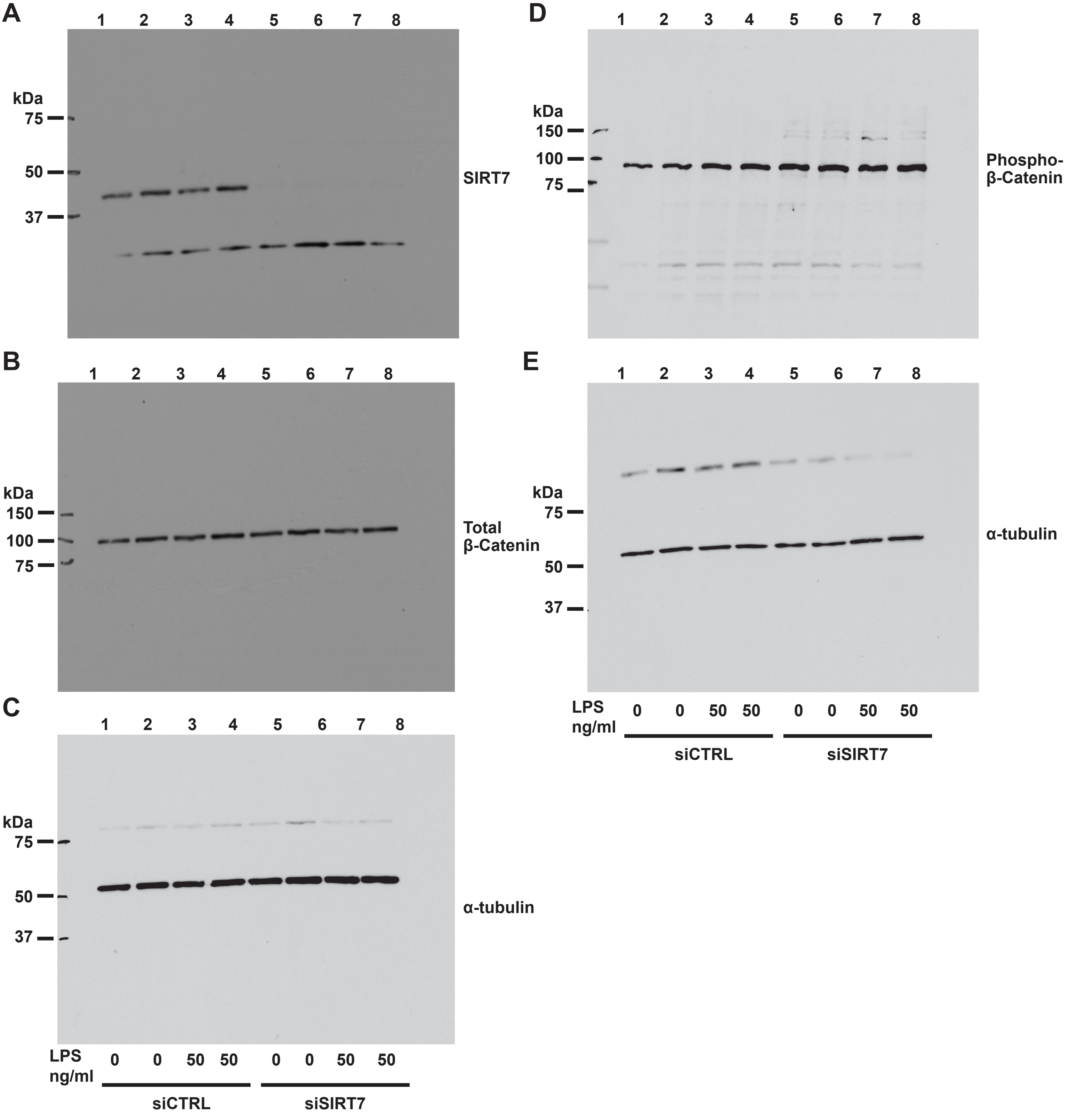

## Supplementary Figure 12

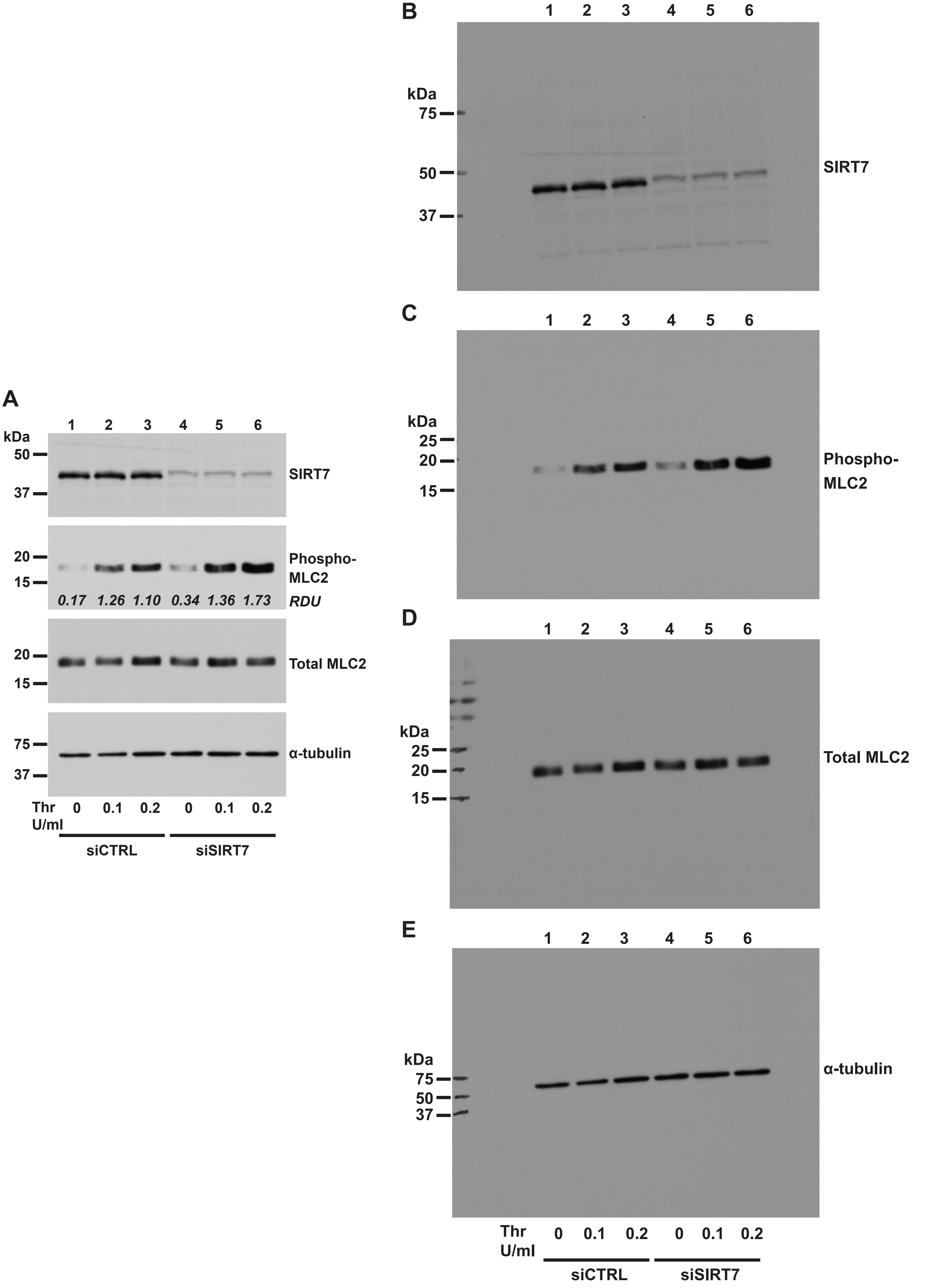

Supplement: Supplementary file 1 — Supplementary Information. [file 41598_2020_69236_MOESM1_ESM.pdf]
